# Massive Hemoptysis Secondary to Rasmussen’s Aneurysm in a Patient With Active Pulmonary Tuberculosis: A Case Report

**DOI:** 10.7759/cureus.99117

**Published:** 2025-12-13

**Authors:** Waqar Farooqi, Nourah Alosaimi, Shaden Alsenaidi, Amanee almalik, Eid Alosaimi

**Affiliations:** 1 Internal Medicine, Faculty of Medicine, Al Maarefa University, Riyadh, SAU; 2 Internal Medicine, King Saud Medical City, Riyadh, SAU; 3 College of Medicine, Al Maarefa University, Riyadh, SAU

**Keywords:** computed tomography pulmonary angiography, endovascular embolization, massive hemoptysis, pseudoaneurysm, pulmonary tuberculosis, rasmussen’s aneurysm

## Abstract

Rasmussen’s aneurysm is a rare but potentially deadly pseudoaneurysm of a pulmonary arterial branch adjacent to a tuberculous cavity. This report describes the case of a 38-year-old Bangladeshi man with uncontrolled type 2 diabetes who presented with sudden massive hemoptysis following six months of cough, weight loss, fever, and pleuritic chest pain. Imaging showed bilateral cavitary pulmonary tuberculosis with a pseudoaneurysm of the left lower-lobe pulmonary artery, consistent with a ruptured Rasmussen’s aneurysm. Urgent endovascular embolization achieved complete hemostasis. This case exemplifies the paramount importance of timely recognition and prompt therapeutic intervention in reducing mortality arising from tuberculosis-associated pulmonary hemorrhage.

## Introduction

Massive hemoptysis can be a life-threatening hemoptysis that results in hypotension, respiratory failure, or airway blockage is a suitable definition of massive hemoptysis. Asphyxiation, not hemorrhagic shock, is the cause of death for patients with this illness [1]. It has been described in a variety of ways depending on factors including the amount of bleeding per hour, the total amount of bleeding per 24 hours, or the existence of aberrant gas exchange or hemodynamic instability. Although there is no agreement, bleeding rates greater than 100 mL/hour or total volumes greater than 500 mL in a 24-hour period are generally regarded as life-threatening hemoptysis. Smaller volumes (50 mL) of hemoptysis may be fatal, depending on the patient's underlying cardiopulmonary condition [2].

If not managed expediently, the mortality rate goes up to 50%. A Rasmussen’s aneurysm represents a false aneurysm of the pulmonary artery secondary to chronic inflammatory erosion and the weakening of the arterial wall adjacent to a tuberculous cavity [3-5]. Predominantly in endemic zones, pulmonary tuberculosis (TB) continues to be the primary etiology [6]. Computed tomography pulmonary angiogram (CTPA) is the most reliable diagnostic modality for assessing the origin of bleeding and confirming the existence of vascular lesions like pseudoaneurysms [7-9]. Endovascular embolization has shown high success rates and significantly reduces mortality compared to surgery [10-12] and thus remains the preferred therapeutic procedure. 

## Case presentation

A 38-year-old Bangladeshi male ex-smoker (three months prior), with poorly controlled type 2 diabetes mellitus, arrived at the emergency department on October 17, with continuous massive hemoptysis for >1 hour. He had six months of productive whitish cough, subjective fever, night sweats, anorexia, and >20 kg weight loss. One month prior to presentation, he developed post-prandial vomiting, watery diarrhea, and left-sided pleuritic chest pain. There was no prior history of tuberculosis, hemoptysis, or anti-tuberculous therapy reported. He was cachectic and pale but alert on examination (Glasgow Coma Scale 15/15).

**Figure 1:**
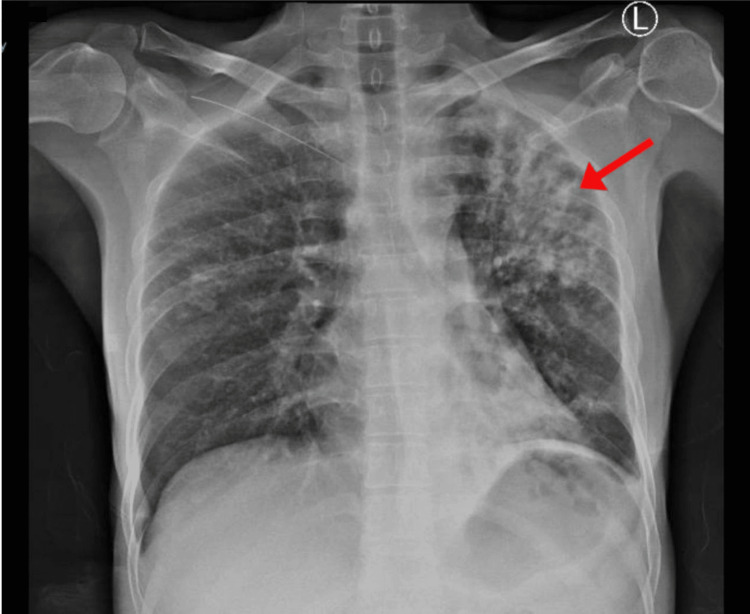
Chest X-ray demonstrated dense left upper-zone consolidation with a subclavicular cavities.

Vitals were as follows: blood pressure 111/78 mmHg, heart rate 102/minute, peripheral oxygen saturation 85%, maintained on 2 L nasal cannula 97%, afebrile (occasional spike to 39.5 °C). Chest auscultation found coarse crackles and wheezes, more prominent on the left. Cardiovascular and abdominal examinations were unremarkable. On October 18, he was transferred to the intensive care unit (ICU) and placed in an isolation bed. At 13:00, he received three additional units of packed red blood cells (PRBCs), with two more units kept on standby. Laboratory investigations and microbiological tests showed: Acid-fast bacilli culture was positive, and polymerase chain reaction (PCR) test for *Mycobacterium tuberculosis* and rifampicin resistance (GeneXpert MTB/RIF PCR) confirmed *M. tuberculosis*. HIV Type 1 and Type 2 testing was non-reactive. Respiratory viral testing, including SARS-CoV-2 virus and a full respiratory PCR panel, detected no pathogens. Urine microscopy showed <10 squamous epithelial cells/low power field (LPF) and >25 neutrophils/LPF. revealed white blood cell count 39×10^9^/L (neutrophilic), hemoglobin 8.7 g/dL (mean corpuscular volume 72 fL), and platelets 652×10^9^/L. Renal and liver function tests were within normal range. Chest X-ray showed dense left upper-zone consolidation with a cavity (Figure 1).

At 20:00 on October 18, he underwent interventional radiology-guided embolization. The initial angiogram of the left pulmonary artery showed left sided small pulmonary artery branch pseudoaneurysm. The decision was made to go with super selection and do embolization using permanent coil material, which was done successfully. No more pseudoaneurysms appeared.

**Figure 2:**
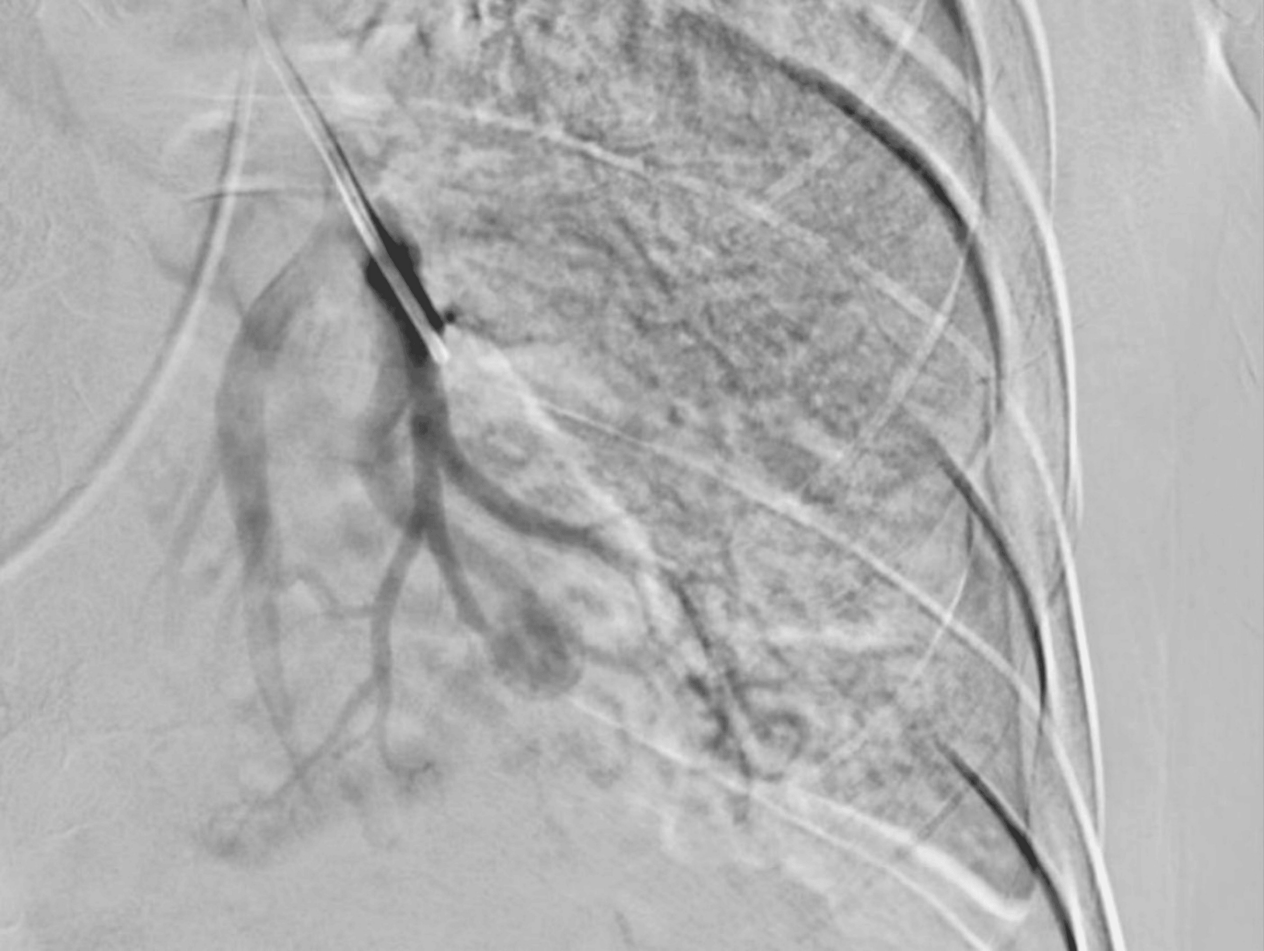
Selective angiogram of the right pulmonary artery reveals a focal saccular outpouching that is consistent with a Rasmussen aneurysm, along with a noticeably hypertrophied pulmonary artery and parenchymal hypervascularity. Before embolization, the image shows active contrast extravasation into the cavitary lesion in the left upper lobe.

The patient was observed for signs of hemoptysis again. No further evidence of pseudoaneurysm formation was observed on subsequent imaging. Manual compression was done after the removal of the right common femoral artery (CFA) and common femoral vein (CFV). The patient received blood transfusions, empirical anti-tuberculous therapy, and intravenous antibiotics. Due to ongoing hemoptysis, he underwent successful interventional radiology-guided embolization of the left pulmonary artery pseudoaneurysm (Figure 2).

CT showed bilateral cavitary consolidation with a left lower-lobe pseudoaneurysm consistent with Rasmussen’s aneurysm (Video 1). No pulmonary embolism was noted. A massive hemoptysis protocol was started, and the patient was given oxygen, three units of PRBCs, and anti-tuberculosis therapy. Urgent interventional radiology-guided embolization of the left pulmonary artery pseudoaneurysm achieved complete hemostasis.

**Video 1:** Axial lung-window CT pulmonary angiogram demonstrates bilateral cavitary lesions and a pseudoaneurysm in a subsegmental branch of the left lower lobe pulmonary artery, consistent with a Rasmussen aneurysm. The lung window setting allows clearer visualization of the cavitary morphology and surrounding parenchymal damage.

**Figure 3:**
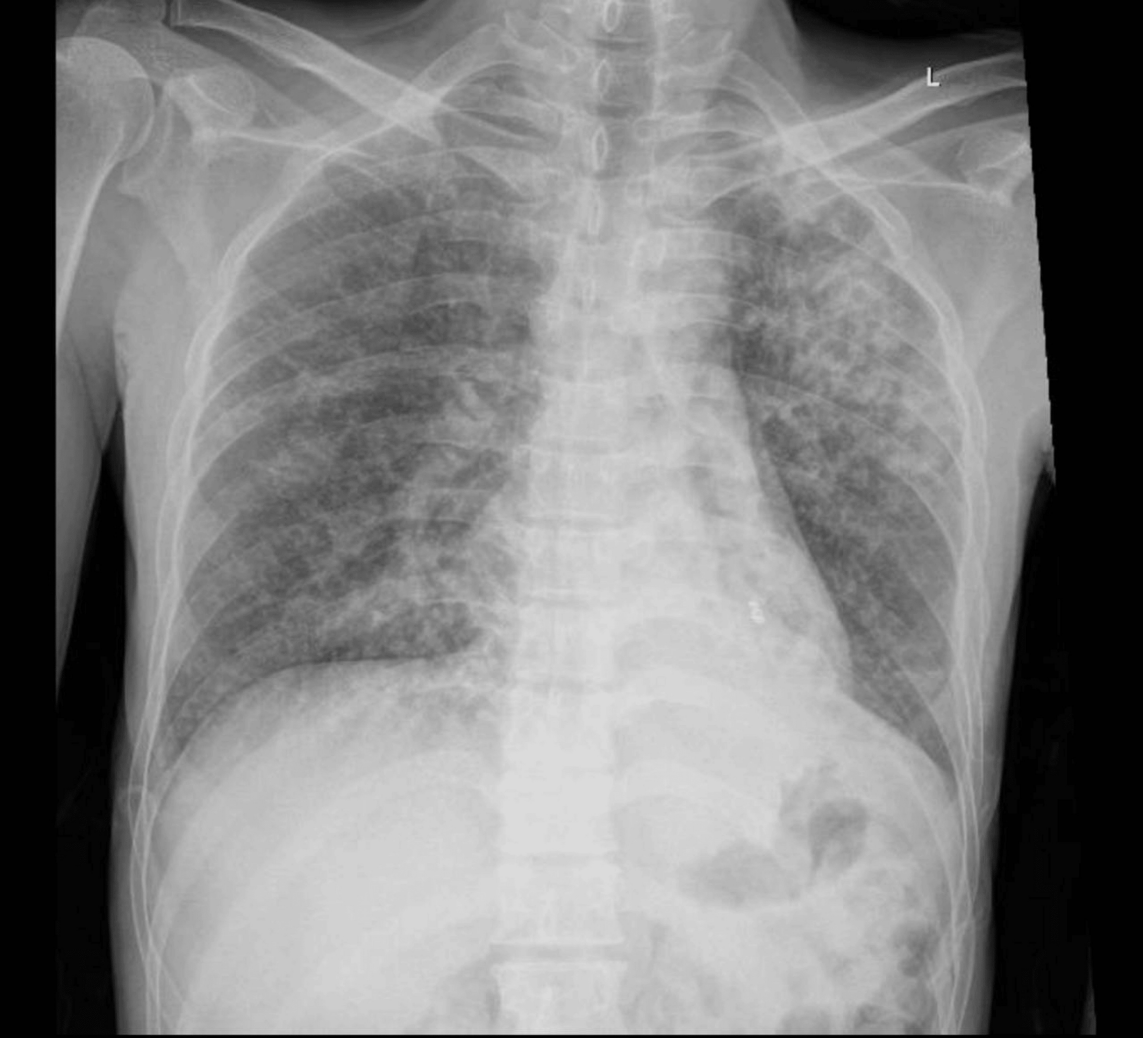
Chest x-ray demonstrated dense left upper-zone consolidation with a subclavicular cavity and marked interval improvement compared with previous radiographs.

On October 19, the patient was awake, alert, maintaining adequate oxygen saturation on room air, and remained hemodynamically stable. Anti-tuberculous therapy was continued as planned. Thoracic surgery documented ongoing ICU management. He stayed in the ICU until October 23, after which he showed dramatic clinical improvement and was transferred to the general ward on October 24. During hospitalization, follow-up imaging on October 30 demonstrated dense left upper-zone consolidation with a subclavicular cavity and marked interval improvement compared with previous radiographs (Figure 3).

He remained hospitalized until November 3, when he was discharged with a structured TB management plan, including an intensive phase from October 20, 2025, to December 20, 2025, and a continuation phase from December 21, 2025, to April 21, 2026, along with follow-up through the mobile tuberculosis team, tuberculosis culture and sensitivity monitoring, routine complete blood count tests, liver and kidney function tests, and the virtual pulmonary tuberculosis clinic.

## Discussion

Rasmussen’s aneurysm is an uncommon and life-threatening complication of pulmonary tuberculosis because of progressive weakening of the pulmonary arterial wall next to a tuberculous cavity. Pseudoaneurysm formation and eventual rupture with inflammatory destruction of the tunica media and adventitia, as well as caseous necrosis, arise [1-3]. In a series of 1114 autopsies of patients with chronic cavitary tuberculosis, Auerbach reported arterial erosion or aneurysmal dilatation in only 45 (4%) [5]. The incidence of rupture is now rare, with the risk estimates being less than 5%, largely ascribed to early diagnosis and effective anti-tuberculous therapy [5,6]. 

Hemoptysis is the primary symptom of Rasmussen’s aneurysm, which may be mild to considerable and fatal. Although it typically occurs unexpectedly, there is a lack of systemic classical tuberculosis symptoms, so the imaging evaluation has proven essential for diagnosing it [7,8]. Coils, n-butyl cyanoacrylate glue, or vascular plugs are used for embolization techniques for delivery of effective hemostasis and preservation of lung parenchyma. All other types of patients are only candidates for surgical intervention if they have failed endovascular therapy, or if they already have adequate embolization and present with recurrent bleeding [9,10].

Chest radiography can capture potentially nonspecific opacities or cavities, but CTPA is the gold standard for identifying pseudoaneurysms and localizing the source of the bleeding. Endovascular embolization has become the leading therapeutic modality, and early bleeding control is reported to be over 90% and results in a marked reduction in mortality compared to surgical resection. For favorable outcomes, multidisciplinary management with interventional radiology, pulmonology, and infectious disease teams is vital. Early detection and early embolization of Rasmussen’s aneurysm are still vital for lowering the mortality and recurrence rates [11,12].

## Conclusions

Rasmussen’s aneurysm should be suspected in any tuberculosis patient presenting with massive hemoptysis, particularly when visual scans reveal cavitary lesions alongside pulmonary arteries. Prompt CTPA evaluation and interventional embolization are life-saving. This case highlights the need for early identification and multidisciplinary collaboration to prevent lethal outcomes.
